# Kinematic Analysis of Mae-Geri Kicks in Beginner and Advanced Kyokushin Karate Athletes

**DOI:** 10.3390/ijerph16173155

**Published:** 2019-08-29

**Authors:** Monika Błaszczyszyn, Agnieszka Szczęsna, Magdalena Pawlyta, Maciej Marszałek, Dariusz Karczmit

**Affiliations:** 1 Faculty of Physical Education and Physiotherapy, Opole University of Technology, 45-758 Opole, Prószkowska 76, Poland; 2 Institute of Informatics, Silesian University of Technology, 44-100 Gliwice, Akademicka 16, Poland; 3 Polish-Japanese Academy of Information Technology, 02-008 Warsaw, Koszykowa 86, Poland; 4 Kyokushin Karate Club, 44-121 Gliwice, Czwartaków 18, Poland; 5 Kyokushin Karate Club, 48-304 Nysa, Bolesława Prusa 14, Poland

**Keywords:** martial art, kinematic analysis, body segments, movement patterns

## Abstract

*Background*: Each of the techniques used in sport is a complex technique requiring a combination of neuromuscular conduction, motor anticipation, and extremely developed proprioception. This is especially the case in martial arts when we deal with a kick or a blow to a specific target. *Methods*: The main purpose of this study was to determine the kinematic differences in the tested movement pattern among athletes with different levels of advancement in the conditions of kicking: in the air, at a target (a shield), and in direct contact with a competitor. Comparative analysis was performed among 26 players: 13 advanced (group G1) and 13 beginners (group G2). Kinematic data was recorded using an optical motion capture system. The examination consisted of performing three tests of mae-geri kick in sequences of three kicks in three different conditions (without a target, with a static target, and with an opponent). The examination was performed with the back leg and only the moment of kick was analyzed. *Results*: The most significant differences were observed in the movement of head, torso, hip, knee, and ankle segments, especially during a kick at a shield. Based on the conducted analysis, we can assume that karate training changes the strategy of neuromuscular control, promoting improvement of mobility pattern efficiency. *Conclusion*: Acquiring this type of knowledge can lead to better results, elimination of errors in training, especially in the initial period of training, and the prevention of possible injuries that occur during exercise or competition.

## 1. Introduction

Karate as a martial art has an enormous stock of techniques. Taking into account that karateka efficiently uses arms and forearms and that the most dangerous weapons are the lower limbs, the stock of technical solutions for the existing situation in the clash with the opponent seems to be almost unlimited. Sport, treated as a relatively safe test of skills, limits the amount of possible techniques, and those techniques that can permanently hurt the opponent have been rejected [1,2,3]. As a rule, athletes use a dozen of the most popular technical solutions, enriching them with many variant forms.

This paper focuses on the kinematic analysis of the mae-geri technique, often used by athletes as a technique introducing or preceding another technique, so mae-geri rarely occurs as a technique ending a fight [3,4]. Mae-geri is used as a ballistic operation when trying to reach the target or the opponent’s body with the help of a foot. The condition of effectiveness is to execute this technique as quickly as possible, mainly in competitions where time and space parameters can cause limited anticipation by the opponent. The effectiveness of the technique requires a dynamic sequence of movement involving the trunk, hip, knee, ankle, and foot, enabling the synergy flowing from the hip to the foot as a result of the activation of the neuromuscular support of the lower limb [5]. In this sequence of movement anticipation from the proximal to the distal segment, it is not clear at which point the movement of the lower limbs is initiated, consisting of rapid hip flexion, knee extension, and plantar flexion of the ankle, leading to extension of the kicking leg [6]. Each of the techniques used in sport is a complex technique requiring connection of neuromuscular conduction, motor anticipation, and extremely developed proprioception [7,8,9]. Particularly, in martial arts, when we deal with a kick or blow to a specific target, the knowledge and understanding of the motor task must be broadly considered which allows one to increase the efficiency of the given pattern in practice. Several studies available in the literature concerning the front kick focus on repeatability, in the kinetic and kinematic structure of the lower limb task or in neuromuscular control in athletes at various stages of training, which suggests that such indicators can be used in sports selection [10,11,12]. 

Acquiring this type of knowledge may lead to better results, elimination of errors in training, especially in the initial period of training, and prevention of possible injuries that occur during exercise or competition [13,14,15,16,17]. Moreover, specified differences allow us to understand the significance of kinematic parameters, which should be an important tool for supporting training methods. 

The purpose of this study was to determine the kinematic differences in the examined mae-geri kick depending on the level of advancement of the athletes in the conditions of kicking: in the air, at a target (a shield), and under the pressure of facing an opponent. 

The objectives of this study were: (1) to investigate the three-dimensional kinematics of the whole body joints during a front kick in Kyokushin karate athletes; (2) to investigate differences in the mae-geri kick movement pattern in athletes of different advancement levels in three conditions: a kick in the air, a kick at a shield, and a kick making contact with an opponent.

## 2. Material and Methods

The data was acquired by an optical motion capture system. Motion capture systems (optical, inertial) are basic systems for obtaining data for kinematic analysis in sport [18,19,20,21].

Kinematic data was recorded using a motion tracking system (Vicon Motion Systems Limited, Oxford, UK) sampling at 250 Hz. A set of 39 reflective markers were attached to specific anatomical landmarks according to the Plug-In Gait Full Body marker set (Vicon Documentation). Additional markers were placed on the target.

The data acquisition was carried out in the Human Motion Lab (HML) of Research and Development Center of the Polish–Japanese Academy of Information Technology in Bytom, Poland. The system for data acquisition consisted of the following elements:

- Ten NIR Vicon MX-T40 cameras with 4 MP resolution (2352 × 1728 px) and 10-bit gray scale. The measurement space has the shape of an ellipsoidal cylinder with the height of 3 m and a base with 6.47 m and 4.2 m axes
- Four HD video cameras (DV Basler Pilot piA 1900-32gc).

Each of the participants was recorded by the above-mentioned cameras. Before the recording, athletes performed a standard individual warm up of about 2 min, including mainly stretching exercises. The athletes had to stay in the measurement space zone. The examination consisted of the mae-geri technique in sequences of three kicks in each configuration (air, target, and opponent). Kicks were performed by the back, dominant leg. The first performance of the mae-geri was a training kick in the air (test I). The second test was a kick at a target (test II), i.e., a training shield held by the trainer. The third kick was done under the pressure of an opponent (test III). In test III, after taking the position kumite, the measured athlete performed a kick at any time. During the attack, the opponent took a defensive position. Each test was repeated two times by each participant. Before performing the examined techniques, each athlete was calibrated after putting on the markers according to the standard procedure of the Vicon system. For this purpose, the athlete had to stand in the T position by joining their legs and raising their arms to the side. This sequence of movements was preceded by the proper adoption of the kumite no kamae fighting posture and proceeding with the kicks [3]. The examination was carried out according to the Helsinki Declaration and each of the respondents gave their written consent to participate in the research. In addition, the scope and goal were approved by the Bioethics Committee of the Chamber of Physicians (Resolution No. 237 of 13 December 2016). The research was carried out between March and April 2017.

### 2.1. Signal Processing and Statistical Analysis 

**Figure 1:**
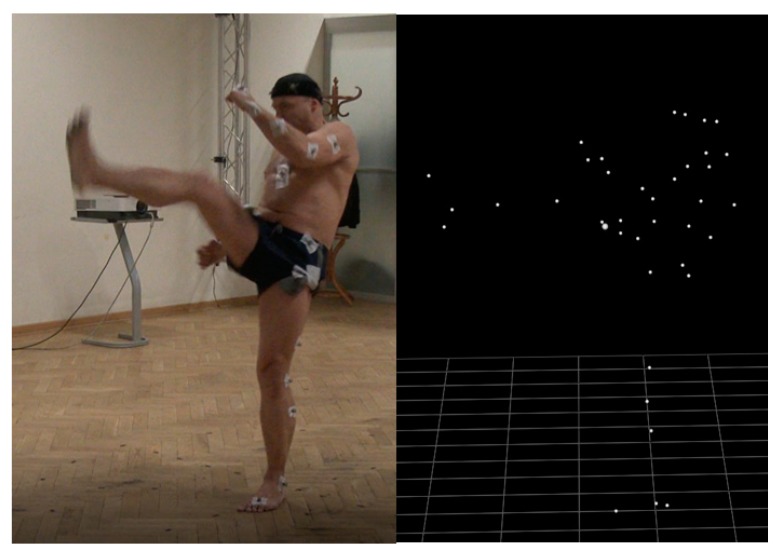
The mae-geri technique into the air with the visualization of tracked markers in the Mokka software.

**Figure 2:**
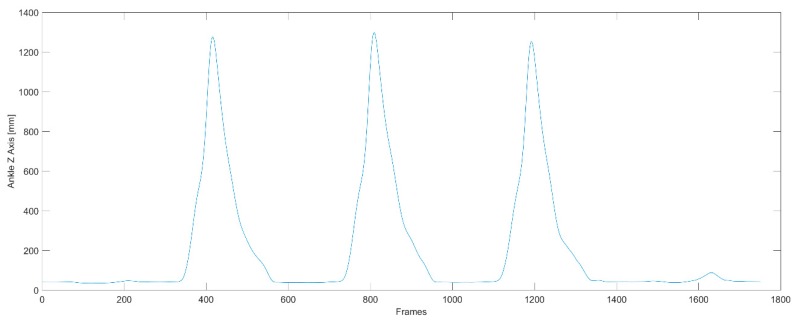
The trajectory of the right ankle (RANK) marker on the Z axis (frontal axis). The three peaks represent the three kicks.

**Figure 3:**
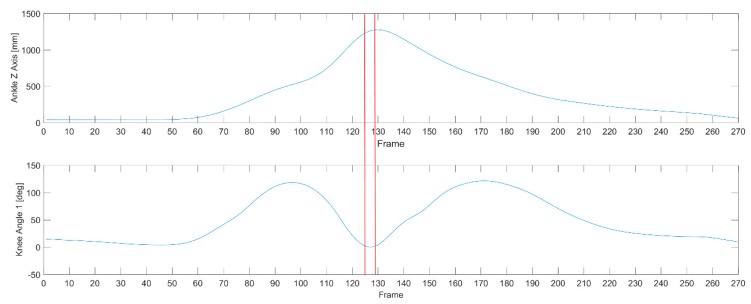
The peak kick phase of separate kick (frames between red lines from 125 and 128) based on ankle marker trajectory (Z axis, frontal) and knee flexion/extension joint angle.

Matlab 2016a with the BTK Toolkit library and the Mokka software were used to analyze the data (Figure 1). The nonparametric Mann–Whitney U-test was used for statistical analysis. The angles of individual joints were determined using the Plug-In Gait software. The separate kick was cut out from recording on the basis of the trajectory of the ankle marker in the frontal axis (z axis) of the kicking leg, thanks to which the kick itself was analyzed (Figure 2). There are three phases of kicking: flexion of kicking limb, peak kick (knee extension), flexion of kicking limb and putting the limb on the floor. Peak kick samples are obtained from the separate kick signal for a minimum value of knee joint flexion angle plus 5 degrees (Figure 3). Such a procedure enables exact cutting of the body pose in the peak kick based on the input signal captured with a frequency of 250 Hz.

The cut-out process is presented in Figure 3. The average time duration of peak phase is as follows:

- Test I—0.016 [s],
- Test II—0.023 [s],
- Test III—0.028 [s].

Mokka software was used for visualization, while all calculations and statistical analyses were carried out in the Matlab 2016a software.

The set of 39 reflective markers the in Plug-In Gait Full Body marker configuration (Vicon Documentation) allows one to obtain 24 joint angles. Rotation angles are represented as three Euler rotations about the axes of particular body segments (rigid body). In the software, the axes are described as follows:

- Transverse axes are those axes which pass from one side of the body to the other;
- Sagittal axes pass from the back of the body to the front; and
- Frontal axes pass in a direction from the base to the apex of body.

In the analysis, the following five joints are used:

- Neck—flexion/extension (angle 1), abduction/adduction (angle 2), external/internal rotation (angle 3).
- Spine—flexion/extension (angle 1), abduction/adduction (angle 2), external/internal rotation (angle 3).
- Hip—flexion/extension (angle 1), abduction/adduction (angle 2), external/internal rotation (angle 3).
- Knee—flexion/extension (angle 1), external/internal rotation (angle 3).
- Ankle—flexion/extension (angle 1), abduction/adduction (angle 2), external/internal rotation (angle 3).

The minimum, maximum, and average angles for a single peak kick phase were determined. These values were averaged over the all kicks of a given participant.

### 2.2. Characteristics of the Participants 

The study concerned 39 athletes, 13 women and 26 men, between 10 and 47 years old and technical grade from 9 kyu to 4 dan, trained in the Kyokushin Karate Club, Gliwice, Poland or Kyokushin Karate Club, Nysa, Poland. Due to the small number of women and the large variation in training experience and technical grade, the group of women was rejected. A group of 26 participants was qualified for the research. Due to technical grade, the athletes were divided into two groups: 13 in each group, group G1—advanced athletes with grade from 2 kyu to 4 dan (brown and black belt), group G2—beginners with grade from 9 kyu to 7 kyu (orange and blue belt).

The characteristics of the respondents are presented in Table 1.

## 3. Results

This work presents kinematic analysis of a kicking pose performed by the participants in three tests: air (test I), shield (test II), and under the pressure of an opponent (test III). A comparative assessment was given to the maximum, minimum, and average values of angles in individual body segments: head (neck), torso (spine), hip, knee, and ankle.

In the head segment movement (Table 2), in test I, statistically significant differences in maximum, minimum, and average values of head flexion were observed. In the frontal plane (lateral movements) significant differences in minimum, average, and maximum values were observed. In G1, the head was placed in the neutral (zero) position with sight directed straight ahead, whereas G2 had a clear movement of the head towards the kicking leg. In test II, the head flexion values were higher in G1 but without statistical significance. The minimum, maximum, and average values of frontal plane movements showed statistical significance and the same tendency as in test I. Additionally, significant differences in rotational head movement occurred in minimum, maximum, and average values. Higher values of rotation towards the kicking leg were observed among the G1 athletes. In test III, higher values of head flexion were still visible in G1; however, these were without statistical significance and values in G2 remained at a similar level as in G1. Minimum, average, and maximum values in the frontal plane (lateral movements) were statistically significant in tests I and II. Among G1 athletes, the head remains in a neutral position with a slight tendency towards abduction, i.e., the opposite to the kicking leg. Among the G2 athletes, a clear adduction movement towards the kicking leg was observed.

In the torso segment movement (Table 3), in test I, statistically significant values were found for the maximum, minimum, and average flexion of the torso (forward tilt). Higher values were obtained by G2 contestants. Statistically significant maximum, minimum, and average values were found in the frontal plane, adduction towards the kicking leg.

In the hip segment movement (Table 4), during tests I, II, and III, statistically significant results were observed in the maximum, minimum, and average values of group G1.

In the knee segment (Table 5), maximum, minimum, and average flexion values were observed as higher among the G2 athletes. Values were similar in test III but without statistical significance, while in test I, the knee was slightly hyperextended.

In the ankle segment in test II (Table 6), significant differences were observed in the maximum, minimum, and average plantar flexion of the foot. In test I and test III, higher values of flexion were observed among G1 in each test but without statistical significance.

## 4. Discussion 

This paper presents the results of a comparative analysis of Kyokushin karate athletes with different levels of advancement. The moment of kicking was analyzed and maximum, minimum, and average angles values of individual body segments—head, torso, hip, knee, ankle—were assessed. According to Portela et al. [4] in the mae-geri technique, the athletes must reach the opponent with the plantar part of the metatarsophalangeal joints, starting with hip flexion movements, knee extension, and plantar ankle bending. This pattern requires keeping a special posture stability while ensuring flexibility of posture for quick withdrawal, adoption of a blocking attitude, or attacking technique. Therefore, the athlete must maintain the appropriate sequence of the kinematic chain. Therefore, we believe that it is not only the position of lower limbs, but also the other body segments that are crucial to ensure the effectiveness of the technique. Correct positioning in the peak kick phase of the head and torso to the kicking leg guarantees not only postural stability, but also provides visual control; however, it requires a special neuromuscular synchronization of all body segments at the right time, especially when the movement is performed at very high speed [22]. For advanced athletes, there is a clear pattern for the mae-geri kick consisting of: energetic head flexion (pulling it to the chest), minimum abduction of the head in the direction opposite to the kicking leg, slight forward flexion of the torso and adduction towards the kicking leg, flexion of the hip, strong adduction and internal rotation, extension of the knee joint, clear plantar flexion of the foot, and slight external rotation. These parameters occurred in all tests. In the beginner athletes, there is no uniform movement pattern; it varies depending on the conditions. The biggest differences were observed in the trajectory of the head. 

Therefore, it can be assumed that the head flexion movement is important and desirable in the assessed technique. Advanced players maintain almost constant values of head flexion irrespective of the test, which indicates the development of a permanent movement pattern for a given segment, which is not observed in beginners who only increased the values of flexion when in contact with an opponent. These differences may indicate a different visual control of advanced athletes and beginners, as well as the lack of proper motor coordination in the evaluated pattern for beginners. Moreover, beginners have a tendency to a deeper forward torso tilt. The knee joint is in flexion in tests II and III. In the air test, the knee is maximally stretched and the position of the foot in the frontal plane is in the neutral position with minimal adduction. In addition, the rotational movements occurring in the hip indicate the direction of internal rotation which forces the knee to be set in the same direction, while the ankle sets in the opposite direction [23,24]. 

Adoption of an appropriate movement pattern is a guarantee of developing appropriate speed and strength and provides a protective attitude, as mentioned by researchers stating that in contact sports, such as karate, capturing moving objects is crucial in successful athletes [25,26]. 

However, the problem of correct capturing of a moving athlete can lead to an infinite number of solutions, because capturing can take place anywhere along the trajectory of the moving object. In addition, a player can use many different movements to achieve the same final capture point. Therefore, the achievement of a proper pattern movement at the initial training stage is not only a guarantee of achieving good results, but also of adequate biomechanics and protection against injuries.

Work in the field of motor control has shown that our brain is trying to simplify the problem, limiting possible movements to those that have been developed and remembered in the nervous system as a permanent movement pattern [27]. Moreover, the brain attempts to adapt to motor activities to optimize the control with energy, while maintaining smoothness and accuracy of movement [28]. Consequently, instead of activation of all joints, the brain modifies the position using only a few joints, leaving less important ones unchanged for effective movement performance [29]. 

The following study limitations can be reported. The number of participants was low and only men were evaluated. The study concerns only the participants’ dominant legs. It is not indicated which athletes have mae-geri as tokui-waza. In the future, it is planned to expand the study by including women, checking kinematic parameters for nondominant legs, and determining the effect of fatigue.

## 5. Conclusions

Based on the conducted analysis, we can assume that karate training changes the strategy of neuromuscular control, promoting the improvement of pattern movement. Thus, to develop appropriate movement activities, trainers must strengthen the acquisition, improvement, and stabilization of neuromuscular movement patterns throughout the kinematic chain, not focusing only on the work of the limbs. Moreover, the development of motor creativity skills should be considered at the earliest possible training stage by contact with a moving target. Acquiring this type of knowledge can lead to better results, elimination of errors in teaching, especially in the initial period of training, and prevention of injuries that occur during exercise or competition.

## Figures and Tables

**Table 1:** Characteristics of the participants.

Variables	G1 (*N* = 13)	G2 (*N* = 13)
Age min/max/x¯/SD (years)	21/47/33.9/8.89	10/12/11.15/0.85
Weight x¯/SD (kg)	73.9/22.72	40.54/6.18
Height x¯/SD (m)	1.74/0.11	1.50/0.06
BMI x¯/SD	23.88/4.04	17.97/2.69

**Table 2:** Comparative analysis of athletes based on neck joint angles.

Variables	Test I			Test II			Test III		
	*p*	Mean G1 (deg)	Mean G2 (deg)	*p*	Mean G1 (deg)	Mean G2 (deg)	*p*	Mean G1 (deg)	Mean G2 (deg)
Neck Ang 1 Max	**0.03** (*)	30.85	23.66	0.07	29.83	21.29	0.81	32.84	31.45
Neck Ang 2 Max	**< 0.001** (***)	−0.22	10.54	**< 0.001** (***)	−1.46	8.45	**< 0.001** (***)	−0.45	7.10
Neck Ang 3 Max	0.16	28.03	30.92	**0.03** (*)	27.16	23.23	0.21	27.90	23.09
Neck Ang 1 Min	**0.02** (*)	30.21	22.84	0.05	28.36	19.58	0.57	31.64	29.60
Neck Ang 2 Min	**< 0.001** (***)	−0.61	10.18	**< 0.001** (***)	−2.15	7.62	**< 0.001** (***)	−1.12	6.53
Neck Ang 3 Min	0.16	27.39	30.21	**0.02** (*)	25.23	20.89	0.16	26.28	21.73
Neck Ang 1 Avg	**0.02** (*)	30.54	23.26	0.07	29.12	20.46	0.70	32.30	30.59
Neck Ang 2 Avg	**< 0.001** (****)	−0.42	10.36	**< 0.01** (**)	−1.81	8.06	**< 0.001** (***)	−0.78	6.83
Neck Ang 3 Avg	0.16	27.72	30.57	**0.03** (*)	26.23	22.12	0.18	27.13	22.44

Table 2 presents: Test I—kick in the air, Test II—kick in a target, Test III—kick under pressure of opponent, G1—advanced players, G2—beginner players, ang—angle joint, min— minimum values, max—maximum values, avg—average values, 1—sagittal plane (flexion/extension), 2—frontal plane (abduction/adduction), 3—transverse plane (rotation external/internal), values, “–” means move to the opposite side (extension, adduction, internal rotation), the degree of significance * *p* < 0.05; ** *p* < 0.01; *** *p* < 0.001.

**Table 3:** Comparative analysis of athletes based on spine joints angles.

Variables	Test I			Test II			Test III		
	*p*	Mean G1 (deg)	Mean G2 (deg)	*p*	Mean G1 (deg)	Mean G2 (deg)	*p*	Mean G1 (deg)	Mean G2 (deg)
Spine Ang 1 Max	**0.01** (*)	22.06	30.14	**< 0.001** (***)	15.71	30.03	**< 0.001** (***)	15.87	25.74
Spine Ang 2 Max	**0.01** (*)	18.92	15.95	**0.04** (*)	11.74	8.83	**< 0.01** (**)	13.60	8.36
Spine Ang 3 Max	0.66	13.81	12.50	0.67	13.74	14.02	0.87	12.53	13.14
Spine Ang 1 Min	**0.01** (*)	21.19	29.02	**< 0.001** (***)	14.81	28.63	**< 0.001** (***)	13.79	24.23
Spine Ang 2 Min	**0.01** (*)	18.47	15.39	**0.03** (*)	11.13	7.90	**<0.01** (**)	12.22	7.13
Spine Ang 3 Min	0.56	13.32	11.88	0.80	12.70	12.64	0.36	10.97	12.33
Spine Ang 1 Avg	**0.01** (*)	21.61	29.57	**< 0.001** (***)	15.25	29.36	**< 0.001** (***)	14.82	25.00
Spine Ang 2 Avg	**0.01** (*)	18.69	15.66	**0.03** (*)	11.43	8.35	**< 0.01** (**)	12.91	7.73
Spine Ang 3 Avg	0.60	13.57	12.20	0.76	13.23	13.36	0.66	11.75	12.76

Table 3 presents: Test I—kick in the air, Test II—kick in a target, Test III—kick under pressure of opponent, G1—advanced players, G2—beginners players, ang—angle joint, min—minimum values, max—maximum values, avg—average values, 1—sagittal plane (flexion), 2—frontal plane (abduction), 3—transverse plane (rotation external), the degree of significance * *p* < 0.05; ** *p* < 0.01; *** *p* < 0.001.

**Table 4:** Comparative analysis of athletes based on hip angles.

Variables	Test I			Test II			Test III		
	*p*	Mean G1 (deg)	Mean G2 (deg)	*p*	Mean G1 (deg)	Mean G2 (deg)	*p*	Mean G1 (deg)	Mean G2 (deg)
Hip Ang 1 Max	0.07	67.05	59.03	0.20	54.94	60.24	0.41	53.40	56.73
Hip Ang 2 Max	**0.03** (*)	−33.62	−27.14	**0.02** (*)	−29.46	−22.64	**< 0.01** (**)	−25.40	−18.30
Hip Ang 3 Max	0.65	−0.02	3.25	0.39	−4.67	3.91	0.31	−5.08	−0.34
Hip Ang 1 Min	0.06	65.66	57.49	0.17	53.25	58.71	0.50	49.76	53.02
Hip Ang 2 Min	0.05	−34.20	−28.59	**0.02** (*)	−31.33	−25.21	**< 0.01** (**)	−27.29	−20.26
Hip Ang 3 Min	0.73	−3.96	−4.86	0.73	−7.92	−2.52	0.61	−10.13	−7.26
Hip Ang 1 Avg	0.06	66.27	58.16	0.17	53.91	59.26	0.43	51.39	54.76
Hip Ang 2 Avg	**0.03** (*)	−33.92	−27.87	**0.02** (*)	−30.43	−24.01	**< 0.01** (**)	−26.36	−19.31
Hip Ang 3 Avg	0.99	−2.21	−1.06	0.56	−6.41	0.74	0.49	−7.68	−4.13

Table 4 presents: Test I—kick in the air, Test II—kick in a target, Test III—kick under pressure of opponent, G1—advanced players, G2—beginners players, ang—angle joint, min—minimum values, max—maximum values, avg—average values, 1—sagittal plane (flexion/extension), 2—frontal plane (abduction/adduction), 3—transverse plane (rotation external/internal), values, “–” means move to the opposite side (extension, adduction, internal rotation), the degree of significance * *p* < 0.05; ** *p* < 0.01; *** *p* < 0.001.

**Table 5:** Comparative analysis of athletes based on knee angles.

Variables	Test I			Test II			Test III		
	*p*	Mean G1 (deg)	Mean G2 (deg)	*p*	Mean G1 (deg)	Mean G2 (deg)	*p*	Mean G1 (deg)	Mean G2 (deg)
Knee Ang 1 Max	0.65	−1.70	−2.20	**0.02** (*)	3.65	12.12	0.30	6.08	11.11
Knee Ang 3 Max	0.25	−9.04	−13.77	0.49	−8.08	−7.52	0.59	−5.88	−10.29
Knee Ang 1 Min	0.68	−3.17	−3.50	0.05	2.09	9.98	0.36	4.27	9.00
Knee Ang 3 Min	0.12	−11.39	−17.19	0.61	−10.08	−10.18	0.66	−9.56	−14.33
Knee Ang 1 Avg	0.69	−2.61	−2.99	**0.04** (*)	2.63	10.67	0.32	4.84	9.64
Knee Ang 3 Avg	0.20	−10.29	−15.60	0.58	−9.31	−9.12	0.61	−7.87	−12.48

The Table 5 presents: Test I—kick in the air, Test II—kick in a target, Test III—kick under pressure of opponent, G1—advanced players, G2—beginner players, ang—angle, min—minimum values, max—maximum values, avg—average values, 1—sagittal plane (flexion/extension), 3—transverse plane (rotation external/internal), values, “–” means move to the opposite side (extension, internal rotation), the degree of significance * *p* < 0.05; ** *p* < 0.01; *** *p* < 0.001.

**Table 6:** Comparative analysis of athletes based on ankle angles.

Variables	Test I			Test II			Test III		
	*p*	Mean G1 (deg)	Mean G2 (deg)	*p*	Mean G1 (deg)	Mean G2 (deg)	*p*	Mean G1 (deg)	Mean G2 (deg)
Ank Ang 1 Max	0.21	−34.23	−27.54	**< 0.001** (***)	−29.86	−15.82	0.59	−23.40	−20.51
Ank Ang 2 Max	0.86	−0.01	0.48	0.09	−0.20	1.73	0.68	0.34	0.22
Ank Ang 3 Max	0.68	3.83	4.49	0.21	5.28	2.00	0.89	4.13	4.23
Ank Ang 1 Min	0.25	−35.66	−29.04	**< 0.001** (***)	−32.78	−19.50	0.73	−26.14	−24.02
Ank Ang 2 Min	0.65	−0.56	−0.75	0.28	−1.01	−0.03	0.99	−0.63	−1.06
Ank Ang 3 Min	0.81	1.81	0.24	0.11	2.07	−3.63	0.64	0.52	−0.12
Ank Ang 1 Avg	0.27	−34.96	−28.31	**< 0.001** (***)	−31.66	−17.96	0.70	−24.90	−22.29
Ank Ang 2 Avg	0.82	−0.32	−0.20	0.15	−0.67	0.78	0.81	−0.18	−0.49
Ank Ang 3 Avg	0.87	2.96	2.59	0.15	3.90	−0.61	0.89	2.43	2.29

The Table 6 presents: Test I—kick in the air, Test II—kick in a target, Test III—kick under pressure of opponent, G1—advanced players, G2—beginner players, ang—angle joint, min—minimum values, max— maximum values, avg—average values, 1—sagittal plane (flexion/extension), 2—frontal plane (abduction/adduction), 3—transverse plane (rotation external/internal), values, “–” means move to the opposite side (extension, adduction, internal rotation), the degree of significance * *p* < 0.05; ** *p* < 0.01; *** *p* < 0.001.
